# Genetic alterations in gastric cancer patients according to sex

**DOI:** 10.18632/aging.202142

**Published:** 2020-12-03

**Authors:** Li-Wen Hsu, Kuo-Hung Huang, Ming-Huang Chen, Wen-Liang Fang, Yee Chao, Su-Shun Lo, Anna Fen-Yau Li, Chew-Wun Wu, Yi-Ming Shyr

**Affiliations:** 1 Division of General Surgery, Department of Surgery, Taipei Veterans General Hospital, Taipei, Taiwan; 2 School of Medicine, National Yang-Ming University, Taipei, Taiwan; 3 Center of Immuno-Oncology, Department of Oncology, Taipei Veterans General Hospital, Taipei, Taiwan; 4 National Yang-Ming University Hospital, Yilan, Taiwan; 5 Department of Pathology, Taipei Veterans General Hospital, Taipei, Taiwan

**Keywords:** gastric cancer, sex, clinicopathological feature, genetic alteration, prognosis

## Abstract

To date, few reports have investigated the genetic alterations and clinicopathological features in gastric cancer (GC) according to sex. In total, 2673 GC patients receiving curative surgery were enrolled. Among the 2673 GC patients, 1979 (74.0%) patients were male. After propensity-score matching, 846 patients were enrolled for the analysis, including 423 males and 423 females. There was no significant difference in the clinicopathological features between the sexes. Regarding the initial recurrence pattern, the males were more likely to develop tumor recurrence and liver metastasis than the females, especially in stage III GC. Regarding the molecular analysis, the males had higher *PD-L1* expression than the females, especially in stage III GC. In addition, the patients aged ≥ 65 years had higher *PD-L1* expression than the patients younger than 65 years. The multivariate analysis demonstrated that sex was among the independent prognostic factors affecting overall survival (OS) and disease-free survival (DFS). Among the patients with liver metastases, *PD-L1* expression was more common among the aged male patients. The males were associated with more tumor recurrence and higher *PD-L1* expression than the females, especially in stage III GC. For GC patients with liver metastases, *PD-L1* testing is recommended, especially among aged male patients.

## INTRODUCTION

Gastric cancer (GC) is the sixth most common cancer and the second most common cause of cancer-related deaths worldwide [1]. Surgical resection is the only curative treatment for GC. The most frequent age of GC onset is between 50 and 70 years. The factors associated with gastric carcinogenesis include Helicobacter pylori infection, Epstein-Barr virus infection, genetic mutations, and microsatellite instability (MSI). [2, 3].

A male predominance in upper gastrointestinal tract cancers, including esophageal cancer and GC, is observed in almost all populations, and the male gender is a well-established risk factor [4, 5]. Some possible reasons contributing to the different incidences between males and females include the protective effects of hormones in females, differences in body iron storage, and different dietary habits and lifestyles [6]. However, strong evidence explaining this difference in incidence is lacking.

It has been reported that in young GC patients, MSI is more common in females than males [7]. However, there is no significant difference in genetic mutations in *hMLH3*, *BRAF*, and *KRAS* between the sexes in young GC patients [7]. Programmed death-ligand 1 (*PD-L1*) expression is reportedly more common in males GC patients [8, 9]; however, a meta-analysis study showed no difference in *PD-L1* expression between the sexes in GC [10]. In addition, other common GC-related genetic alterations exist, including *PIK3CA* amplifications and genetic mutations in the *PI3K/AKT* pathway, *TP53*, and *ARID1A* [3]. To date, whether differences exist in common GC-related genes between the sexes is still unclear.

The aim of the current study was to compare the clinicopathological features, recurrence patterns, prognoses, and genetic alterations in GC between the sexes after curative surgery.

## RESULTS

### Clinicopathological features

Among the 2673 GC patients, 1979 (74.0%) were males. As shown in Table 1, the males were more likely to be older and have more tumors located in the upper stomach, more Borrmann type 3&4 tumors, more intestinal-type tumors, more lymphovascular invasion, more advanced pathological T category, and more pathological Tumor, Node, Metastasis (TNM) stage III tumors than the females.

**Table 1:** Clinical profile in GC patients before and after propensity matching according to different genders.

**Variables**	**Before propensity score matching**				**After propensity score matching**		
	**Male n=1979 n (%)**	**Female n=694 n (%)**	***P* value**		**Male n=423 n (%)**	**Female n=423 n (%)**	***P* value**
Age (years old)			**<0.001**				0.945
<65	551 (27.8)	402 (57.9)			185 (43.7)	186 (44.0)	
≥65	1428 (72.2)	292 (42.1)			238 (56.3)	237 (56.0)	
Tumor size (cm)			0.053				0.729
<5	1042 (52.7)	395 (56.9)			232 (54.8)	237 (56.0)	
≥5	937 (47.3)	299 (43.1)			191 (45.2)	186 (44.0)	
Tumor location			**<0.001**				0.668
Upper stomach	372 (18.8)	86 (12.4)			49 (11.6)	60 (14.2)	
Middle stomach	676 (34.2)	306 (44.1)			165 (39.0)	164 (38.8)	
Lower stomach	876 (44.3)	290 (41.8)			201 (47.5)	193 (45.6)	
Whole stomach	55 (2.8)	12 (1.7)			8 (1.9)	6 (1.4)	
Gross appearance			**<0.001**				0.692
Superficial type	696 (35.2)	313 (45.1)			181 (42.8)	183 (43.3)	
Borrmann type 1	90 (4.5)	32 (4.6)			11 (2.6)	23 (5.4)	
Borrmann type 2	321 (16.2)	103 (14.8)			63 (14.9)	61 (14.4)	
Borrmann type 3	730 (36.9)	190 (27.4)			142 (33.6)	119 (28.1)	
Borrmann type 4	142 (7.2)	56 (8.1)			(6.1)	37 (8.7)	
Lauren’s classification			**<0.001**				0.449
Intestinal type	1265 (63.9)	283 (40.8)			226 (53.4)	215 (50.8)	
Diffuse type	714 (36.1)	411 (59.2)			197 (46.6)	208 (49.2)	
Lymphovascular invasion	1152 (58.2)	353 (50.9)	**0.001**		222 (52.5)	216 (51.1)	0.680
Lymphoid stroma	188 (9.5)	51 (7.3)	0.087		31 (7.3)	32 (7.6)	0.896
Adjuvant chemotherapy	166 (8.4)	82 (11.8)	**0.007**		40 (9.5)	42 (9.9)	0.816
Pathological T category			**<0.001**				0.074
T1	612 (30.9)	287 (41.4)			153 (36.2)	182 (43.0)	
T2	313 (15.8)	82 (11.8)			65 (15.4)	45 (10.7)	
T3	525 (26.5)	180 (25.9)			101 (23.9)	104 (24.6)	
T4	529 (26.8)	145 (20.9)			104 (24.5)	92 (21.7)	
Pathological N category			0.199				0.825
N0	938 (47.5)	336 (48.4)			209 (49.4)	213 (50.4)	
N1	269 (13.5)	112 (16.1)			66 (15.6)	57 (13.5)	
N2	315 (15.9)	94 (13.6)			59 (13.9)	58 (13.7)	
N3	457 (23.1)	152 (21.9)			89 (21.1)	95 (22.4)	
Pathological TNM Stage			**0.015**				0.622
I	764 (38.6)	295 (42.5)			177 (41.8)	189 (44.7)	
II	428 (21.6)	166 (23.9)			97 (22.9)	87 (20.6)	
III	787 (39.8)	233 (33.6)			149 (35.3)	147 (34.7)	

TNM: tumor, node, metastasis; bold: statistically significant.

As shown in Table 1, after propensity-score matching at a 1: 1 ratio, 846 patients were enrolled for the subsequent analysis, including 423 males and 423 females. There is no significant difference in the clinicopathological features between the males and females (Table 1), and no difference was observed between the sexes in each TNM stage (Table 2).

**Table 2:** Clinical profile in GC patients between different sexes in stage I-III GC.

**Variables**	**Stage I**				**Stage II**				**Stage III**		
	**Male n=177 n (%)**	**Female n=189 n (%)**	***P* value**		**Male n=97 n (%)**	**Female n=87 n (%)**	***P* value**		**Male n=149 n (%)**	**Female n=147 n (%)**	***P* value**
Age (years old)			0.760				0.611				0.825
<65	74 (41.8)	82 (43.4)			46 (47.4)	38 (43.7)			65 (43.6)	66 (44.9)	
>65	103 (58.2)	107 (56.6)			31 (52.6)	49 (56.3)			84 (56.4)	81 (55.1)	
Tumor size (cm)			0.764				0.215				0.425
<5	150 (84.7)	158 (83.6)			48 (49.5)	51 (58.6)			34 (22.8)	28 (19.0)	
>5	27 (15.3)	31 (16.4)			49 (50.5)	36 (41.4)			115 (77.2)	119 (81.0)	
Tumor location			0.926				0.817				0.556
Upper stomach	15 (8.5)	18 (9.5)			12 (12.4)	11 (12.6)			22 (14.8)	31 (21.1)	
Middle stomach	78 (44.1)	84 (44.4)			31 (32.0)	27 (31.0)			56 (37.6)	53 (36.1)	
Lower stomach	84 (47.5)	87 (46.0)			53 (54.6)	49 (56.3)			64 (43.0)	57 (38.8)	
Whole stomach	0	0			1 (1.0)	0			7 (4.7)	6 (4.1)	
Gross appearance			0.599				0.279				0.240
Superficial type	147 (83.1)	150 (79.4)			21 (21.6)	25 (28.7)			13 (8.7)	8 (5.4)	
Borrmann type 1	5 (2.8)	9 (4.8)			3 (3.1)	8 (9.2)			3 (2.0)	6 (4.1)	
Borrmann type 2	14 (5.1)	14 (7.4)			28 (28.9)	22 (25.3)			26 (17.4)	25 (17.0)	
Baromann type 3	15 (8.5)	14 (7.4)			39 (40.2)	27 (31.0)			88 (59.1)	78 (53.1)	
Borrmann type 4	1 (0.6)	2 (1.1)			6 (6.2)	5 (5.7)			19 (12.8)	30 (20.4)	
Lauren’s classification			0.715				0.138				0.460
Intestinal type	111 (62.7)	122 (64.6)			53 (54.6)	38 (43.7)			62 (41.6)	55 (37.4)	
Diffuse type	66 (37.3)	67 (35.4)			44 (45.4)	49 (56.3)			87 (58.4)	92 (62.6)	
Lymphovascular invasion	25 (14.1)	33 (17.5)	0.382		60 (61.9)	49 (56.3)	0.446		137 (91.9)	134 (91.2)	
Lymphoid stroma	11 (6.2)	11 (5.8)	0.874		9 (9.3)	8 (9.2)	0.985		11 (7.4)	13 (8.8)	0.645
Adjuvant chemotherapy	0	0	-		8 (8.2)	8 (9.2)	0.820		32 (21.5)	34 (23.1)	0.733
Pathological T category			0.114				0.086				0.476
T1	145 (81.9)	166 (87.8)			7 (7.2)	16 (18.4)			1 (0.7)	0	
T2	32 (18.1)	23 (12.2)			29 (29.9)	17 (19.5)			4 (2.7)	5 (3.4)	
T3	0	0			43 (44.3)	37 (42.5)			58 (38.9)	67 (45.6)	
T4	0	0			18 (18.6)	17 (19.5)			86 (57.7)	75 (51.0)	
Pathological N category			0.410				0.632				0.458
N0	166 (93.8)	173 (91.5)			40 (41.2)	38 (43.7)			3 (2.0)	2 (1.4)	
N1	11 (6.2)	16 (8.5)			34 (35.1)	25 (28.7)			21 (14.1)	16 (10.9)	
N2	0	0			23 (23.7)	20 (23.0)			36 (24.2)	38 (25.9)	
N3	0	0			0	4 (4.6)			89 (59.7)	91 (61.9)	

### Initial recurrence patterns

The mean follow-up time was 91.9 months, which was similar between males and females (91.5 vs. 92.4 months, *P*=0.875). The longest follow-up time was 344.6 months. Among the 846 patients, 207 patients (24.5%) had tumor recurrence during follow-up; 445 patients died during follow-up, and 49 patients (5.8%) had follow-up time shorter than 3 years; among them, there were 22 stage I diseases, 13 stage II diseases, and 14 stage III diseases. Among the 49 patients, four patients had tumor recurrence, including three patients with stage III diseases and one patient with stage II disease.

As shown in Table 3, the males were more likely to develop tumor recurrence, distant metastases, and liver metastases than the females. In stage I GC, the males were more likely to develop tumor recurrence than the females. In stage II GC, there was no difference in the initial recurrence patterns between the sexes. In stage III GC, the males were more likely to develop tumor recurrence and liver metastases than the females.

**Table 3:** The initial recurrence pattern between different genders in stage I-III GC patients after propensity score matching.

**Initial recurrence pattern**	**All GC**				**Stage I GC**				**Stage II GC**				**Stage III GC**		
	**Male n=423 n (%)**	**Female n=423 n (%)**	***P* value**		**Male n=177 n (%)**	**Female n=189 n (%)**	***P* value**		**Male n=97 n (%)**	**Female n=87 n (%)**	***P* value**		**Male n=149 n (%)**	**Female n=147 n (%)**	***P* value**
Total patients with recurrence	118 (27.9)	89 (21.0)	**0.020**		19 (10.7)	9 (4.8)	**0.032**		19 (19.6)	18 (20.7)	0.852		80 (53.7)	62 (42.2)	**0.047**
Locoregional recurrence	53 (12.5)	38 (9.0)	0.096		8 (4.5)	4 (2.1)	0.197		9 (9.3)	8 (9.2)	0.985		36 (24.2)	26 (17.7)	0.171
Distant metastasis	98 (23.2)	75 (17.7)	**0.030**		11 (6.2)	6 (3.2)	0.167		16 (16.5)	15 (17.2)	0.893		71 (47.7)	54 (36.7)	0.057
Peritoneal dissemination	45 (10.6)	41 (9.7)	0.649		1 (0.6)	3 (1.6)	0.347		7 (7.2)	8 (9.2)	0.624		37 (24.8)	30 (20.4)	0.363
Hematogenous metastasis	61 (14.4)	26 (6.1)	**<0.001**		9 (5.1)	3 (1.6)	0.060		10 (10.3)	5 (5.7)	0.259		42 (28.2)	18 (12.2)	**0.001**
Liver	40 (9.5)	16 (3.8)	**0.001**		4 (2.6)	1 (0.5)	0.154		7 (7.2)	3 (3.4)	0.260		29 (19.5)	12 (8.2)	**0.005**
Lung	7 (1.7)	5 (1.2)	0.561		2 (1.1)	1 (0.5)	0.524		1 (1.0)	1 (1.1)	0.938		4 (2.7)	3 (2.0)	0.716
Bone	13 (3.1)	7 (1.7)	0.175		4 (2.6)	1 (0.5)	0.154		2 (2.1)	2 (2.3)	0.912		7 (4.7)	4 (2.7)	0.369
Brain	5 (1.2)	0	0.062		0	0	-		1 (1.0)	0	0.342		4 (2.7)	0	0.122
Adrenal	1 (0.2)	2 (0.5)	0.563		0	0	-		0	0	-		1 (0.7)	2 (1.4)	0.554
Skin	0	0	-		0	0	-		0	0	-		0	0	-
Distant lymphatic recurrence	23 (5.4)	22 (5.2)	0.878		1 (0.6)	1 (0.5)	0.963		1 (1.0)	4 (4.6)	0.137		21 (14.1)	17 (11.6)	0.515

Some patients had more than one recurrence pattern; bold: statistically significant.

### Survival analysis

**Figure 1:**
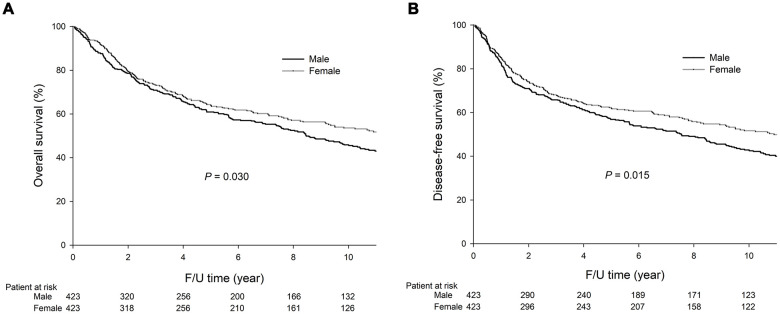
**The 5-year OS (60.9% vs. 64.0%, *P*=0.030) and DFS (56.9% vs. 62.0%, *P*=0.015) rates were significantly lower in the males than the females.** The survival curves are shown as follows: (**A**) OS curves of GC patients; (**B**) DFS curves of GC patients.

**Figure 2:**
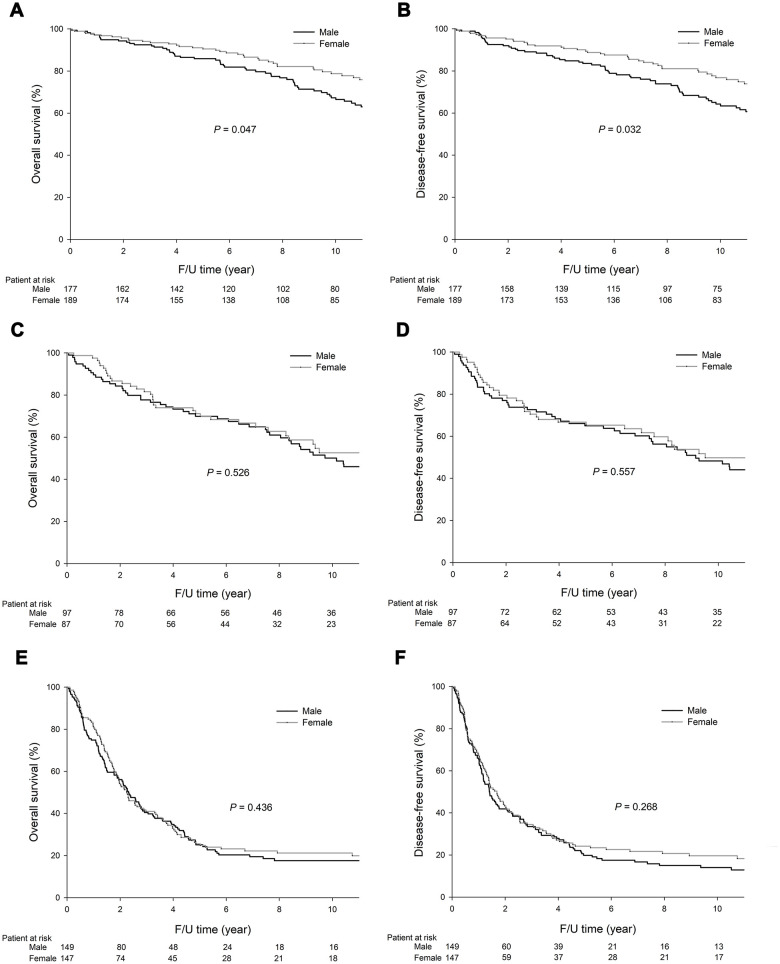
**Among the stage I GC patients, the 5-year OS (85.9% vs. 90.5%, *P*=0.047) and DFS (83.6% vs. 88.8%, *P*=0.032) rates were significantly lower in the males than the females.** Among the stage II GC patients, the 5-year OS (69.9% vs. 71.2%, *P*=0.526) and DFS (65.0% vs. 66.7%, *P*=0.557) rates did significantly differ between the males and females. Among the stage III GC patients, the 5-year OS (25.1% vs. 25.6%, *P*=0.436) and DFS (19.8% vs. 24.2%, *P*=0.268) rates did not significantly differ between the males and females. The survival curves are shown as follows: (**A**) OS curves of stage I GC patients; (**B**) DFS curves of stage I GC patients; (**C**) OS curves of stage II GC patients; (**D**) DFS curves of stage II GC patients; (**E**) OS curves of stage III GC patients; and (**F**) DFS curves of stage III GC patients.

As shown in Figure 1, the males had significantly lower 5-year overall survival (OS) (60.9% vs. 64.0%, *P*=0.030, Figure 1A) and disease-free survival (DFS) (56.9% vs. 62.0%, *P*=0.015, Figure 1B) rates than the females. As shown in Figure 2, in stage I GC, the males had significantly lower 5-year OS (85.9% vs. 90.5%, *P*=0.047, Figure 2A) and DFS (83.6% vs. 88.8%, *P*=0.032, Figure 2B) rates than the females. In stage II GC, there was no significant difference in the OS (69.9% vs. 71.2%, *P*=0.526, Figure 2C) and DFS (65.0% vs. 66.7%, *P*=0.557, Figure 2D) rates between the sexes. In stage III GC, there was no significant difference in the OS (25.1% vs. 25.6%, *P*=0.436, Figure 2E) and DFS (19.8% vs. 24.2%, *P*=0.268, Figure 2F) rates between the sexes.

As shown in Tables 4 and 5, the univariate analysis demonstrated that age, gender, tumor location, gross appearance, adjuvant chemotherapy, lymphovascular invasion, and pathological T and N categories were associated with OS and DFS. The aforementioned eight variables were included in a multivariate Cox proportional hazards model to adjust for the effects of covariates. The multivariate analysis demonstrated that age, gender, gross appearance, and pathological T and N categories were independent prognostic factors affecting OS and DFS.

**Table 4:** Univariate and multivariate analysis of factors affecting OS of GC patients after curative surgery.

**Variables**	**Univariate analysis**				**Multivariate analysis**		
	**HR**	**95%CI**	***P* value**		**HR**	**95%CI**	***P* value**
Age (years old)			**<0.001**				**<0.001**
< 65	1.00				1.00		
≥65	1.78	1.459-2.163			1.90	1.551-2.321	
Gender			**0.030**				**0.042**
Male	1.00				1.00		
Female	0.81	0.675-0.980			0.82	0.674-0.993	
Tumor location			**<0.001**				0.131
Upper stomach	1.00				1.00		
Middle stomach	0.63	0.469-0.841			0.71	0.526-0.961	
Lower stomach	0.76	0.574-0.999			0.82	0.616-1.091	
Whole stomach	2.85	1.568-5.187			1.03	0.542-1.947	
Gross appearance			**<0.001**				**0.003**
Superficial type	1.00				1.00		
Bormann type 1	2.07	1.278-3.351			1.30	0.776-2.176	
Bormann type 2	2.01	1.486-2.716			0.88	0.599-1.292	
Bormann type 3	3.34	2.668-4.183			1.42	1.017-1.978	
Bormann type 4	4.98	3.502-7.067			1.82	1.155-2.873	
Lymphovascular invasion			**<0.001**				0.174
Absent	1.00				1.00		
Present	2.86	2.342-3.495			1.21	0.920-1.591	
Lauren’s classification			0.263				
Intestinal type	1.00						
Diffuse type	1.11	0.923-1.340					
Adjuvant chemotherapy			**<0.001**				0.581
No	1.00				1.00		
Yes	2.00	1.473-2.731			0.91	0.640-1.285	
Pathological T category			**<0.001**				**<0.001**
T1	1.00				1.00		
T2	1.37	0.978-1.912			0.94	0.635-1.396	
T3	3.38	2.619-4.358			1.63	1.099-2.415	
T4	4.53	3.531-5.806			1.89	1.268-2.809	
Pathological N category			**<0.001**				**<0.001**
N0	1.00				1.00		
N1	1.46	1.086-1.957			1.03	0.743-1.425	
N2	2.36	1.775-3.143			1.53	1.106-2.119	
N3	6.67	5.285-8.429			3.47	2.532-4.766	

OS: overall survival; DFS: disease-free survival; bold: statistically significant.

**Table 5:** Univariate and multivariate analysis of factors affecting DFS of GC patients after curative surgery.

**Variables**	**Univariate analysis**				**Multivariate analysis**		
	**HR**	**95%CI**	***P* value**		**HR**	**95%CI**	***P* value**
Age (years old)			**<0.001**				**<0.001**
< 65	1.00				1.00		
≥65	1.75	1.443-2.120			1.85	1.517-2.250	
Gender			**0.015**				**0.018**
Male	1.00				1.00		
Female	0.80	0.664-0.957			0.80	0.659-0.961	
Tumor location			**<0.001**				0.054
Upper stomach	1.00				1.00		
Middle stomach	0.61	0.459-0.812			0.68	0.507-0.915	
Lower stomach	0.74	0.561-0.965			0.84	0.634-1.111	
Whole stomach	2.78	1.534-5.047			0.97	0.513-1.835	
Gross appearance			**<0.001**				**0.004**
Superficial type	1.00				1.00		
Bormann type 1	1.95	1.208-3.155			1.29	0.772-2.142	
Bormann type 2	2.00	1.491-2.686			0.90	0.620-1.306	
Bormann type 3	3.35	2.688-4.174			1.42	1.026-1.963	
Bormann type 4	5.00	3.572-7.006			1.76	1.134-2.737	
Lymphovascular invasion			**<0.001**				0.149
Absent	1.00				1.00		
Present	2.92	2.404-3.556			1.22	0.931-1.602	
Lauren’s classification			0.351				
Intestinal type	1.00						
Diffuse type	1.09	0.909-1.309					
Adjuvant chemotherapy			**<0.001**				0.796
No	1.00				1.00		
Yes	2.27	1.702-3.026			1.05	0.748-1.461	
Pathological T category			**<0.001**				**<0.001**
T1	1.00				1.00		
T2	1.34	0.962-1.859			0.90	0.610-1.322	
T3	3.32	2.588-4.251			1.50	1.021-2.203	
T4	4.67	3.668-5.954			1.95	1.329-2.874	
Pathological N category			**<0.001**				**<0.001**
N0	1.00				1.00		
N1	1.50	1.128-2.006			1.07	0.778-1.476	
N2	2.41	1.821-3.179			1.53	1.116-2.107	
N3	6.91	5.491-8.691			3.55	2.599-4.859	

OS: overall survival; DFS: disease-free survival; bold: statistically significant.

### Analysis of genetic alterations

Genetic alterations were analyzed in 438 GC patients with available GC tissues. As shown in Table 6, the males had higher *PD-L1* expression than the females (31.1% vs. 20.2%, *P*=0.020). Patients aged 65 years or older had higher *PD-L1* expression than those younger than 65 years (33.1% vs. 20.8%, *P*=0.005).

**Table 6:** The molecular differences between genders in GC patients after curative surgery.

	**All GC**				**Stage I GC**				**Stage II GC**				**Stage III GC**		
	**Male n=309 n (%)**	**Female n=129 n (%)**	***P* value**		**Male n=49 n (%)**	**Female n=39 n (%)**	***P* value**		**Male n=90 n (%)**	**Female n=35 n (%)**	***P* value**		**Male n=170 n (%)**	**Female n=55 n (%)**	***P* value**
MSI status			0.355				**0.033**				0.106				0.115
MSI-L/S	284 (91.9)	116 (89.1)			47 (95.9)	32 (82.1)			85 (94.4)	30 (85.7)			152 (89.4)	53 (96.4)	
MSI-H	25 (8.1)	14 (10.9)			2 (4.1)	7 (17.9)			5 (5.6)	5 (14.3)			18 (10.6)	2 (3.6)	
*PD-L1* expression	96 (31.1)	26 (20.2)	**0.020**		14 (28.6)	8 (20.5)	0.386		28 (31.1)	9 (25.7)	0.553		53 (31.2)	9 (16.4)	**0.033**
*PIK3CA* amplification	137 (44.3)	69 (53.5)	0.080		20 (40.8)	19 (48.7)	0.459		39 (43.3)	19 (54.3)	0.270		78 (45.9)	31 (56.4)	0.176
Genetic mutations															
*PI3K/AKT* pathway	54 (17.5)	16 (12.4)	0.182		8 (16.3)	4 (10.3)	0.410		15 (16.7)	5 (14.3)	0.744		31 (18.3)	7 (12.7)	0.335
*TP53*	42 (13.6)	20 (15.5)	0.601		6 (12.2)	7 (17.9)	0.454		11 (12.2)	1 (2.9)	0.111		25 (14.7)	12 (21.8)	0.216
*ARID1A*	42 (13.6)	10 (7.8)	0.085		7 (14.3)	4 (10.3)	0.570		13 (14.4)	2 (5.7)	0.177		22 (12.9)	4 (7.3)	0.253
*BRAF*	2 (0.6)	0	0.360		1 (2.0)	0	0.370		1 (1.1)	0	0.531		0	0	-
*KRAS*	4 (1.3)	6 (4.7)	0.071		2 (4.1)	2 (5.1)	0.815		2 (2.2)	2 (5.7)	0.319		0	2 (3.6)	0.059

MSI: microsatellite instability; MSI-H: MSI-high; MSI-L/S: MSI-low/stable; bold: statistically significant.

In stage I GC, the females were more likely to have MSI-H tumors than the males (17.9% vs. 4.1%, *P*=0.033). In stage III GC, the males were more likely to exhibit *PD-L1* expression than the females (31.2% vs. 16.4%, *P*=0.033). Among the 225 stage III GC, the patients aged 65 years or older were more likely to exhibit *PD-L1* expression than the patients younger than 65 years (29% vs. 14.9%, *P*=0.013).

Among the 438 patients, forty-five (10.3%) patients, including 39 males and 6 females, developed liver metastases. Among these 45 patients with liver metastases, 16 (35.6%) patients had *PD-L1* expression; 14 of these patients (14/16, 87.5%) were males, and 12 of the 14 (85.7%) males were aged 65 years or older.

## DISCUSSION

To minimize selection bias, we performed propensity-score matching and analyzed the clinicopathological feature differences between the sexes in our GC patients. Our results demonstrate that the males are associated with more tumor recurrence, more liver metastasis, and worse prognoses than the females with GC. In addition, the molecular analysis demonstrated that *PD-L1* expression was more common among the aged male patients, especially those with stage III GC. Among the patients with liver metastases, *PD-L1* expression was more common among the aged male patients.

In the present study, sex was one of the independent prognostic factors of OS and DFS. Although gross appearance and pathological T and N categories were significantly related to each other, all of the five independent prognostic factors were still significantly associated with OS and DFS by multivariate analysis. It seems that all the five factors play an important role in GC patient prognosis.

A meta-analysis [11] showed that MSI-H GC was associated with females, an older age, and intestinal-type GC. In the present study, a significantly higher tumor recurrence rate and a significantly higher frequency of MSI-H tumors was observed in the females than the males (17.9% vs. 4.1%, *P*=0.003) in stage I GC only. MSI-H tumors were associated with a better prognosis than MSI-L/S tumors in GC [12]. The reason why the males were associated with a worse prognosis than the females in stage I GC is possibly due to a higher tumor recurrence rate and fewer MSI-H tumors in the males than females. In stage III GC, although the male patients had more tumor recurrence and liver metastases than the female patients, the OS and DFS rates did not significantly differ between the sexes. A possible reason is that the tumor recurrence rates are high in stage III GC, with 53.7% in males and 42.2% in females. In addition, the OS (25.1% vs. 25.6%, *P*=0.436) and DFS (19.8% vs. 24.2%, *P*=0.268) rates were relatively low in both the males and females, resulting in no significant difference in survival rates between the sexes in stage III GC.

Among the 438 patients who underwent molecular analysis in the present study, the males had higher *PD-L1* expression than the females (27.1% vs. 10.9%, *P*=0.014), and the aged patients were associated with higher *PD-L1* expression than the younger patients (29% vs. 14.9%, *P*=0.013). In addition, interestingly, among the GC patients with liver metastases and *PD-L1* expression, most (14/16, 87.5%) patients were males and 12 of the 14 (85.7%) males were aged over 65 years.

*PD-L1* expression has been reported to be higher in metastatic liver tumors than primary gastric tumor cells and tumor infiltrating lymphocytes [13]. Consequently, immunotherapy may be beneficial for GC patients with liver metastases. According to our results, we recommend evaluating *PD-L1* expression in GC patients who develop liver metastases, especially aged male patients, which may provide useful information for evaluating immunotherapy in these patients.

The *PD-L1* CPS score was developed using the 22C3 assay as a companion diagnostic for immunotherapy in GC, which was approved by the U.S. Food and Drug Administration [14]. Most trials regarding the use of immunotherapy in GC are designed for metastatic GC patients. A randomized, multicenter, double-blind, placebo-controlled phase 3 study, ATTRACTION-05 (ONO-4538-38/BMS CA209844), investigating the role of Nivolumab in combination with adjuvant chemotherapy in pathological stage III gastric and esophagogastric junction cancer has completed patient enrollment, and the results are pending. The results of the above trial may provide useful information regarding the selection of patients who can benefit from the use of immunotherapy and chemotherapy in adjuvant setting. In the present study, *PD-L1* expression was more common in male and aged patients, especially in stage III GC, who may be beneficial for adjuvant immunotherapy.

Whole-genome sequencing demonstrated that males had more somatic structural variants than females in GC, but the underlying reason and clinical significance for this result is unclear [15]. Chromosome conformation capture (Hi-C) methods have identified subchromosomal structures of higher-order chromatin interactions called topologically associated domains (TADs) that are separated from each other by boundary regions [16, 17]. By subdividing the genome into discrete regulatory units, TADs restrict the contacts that enhancers establish with their target genes [18–20]. One of the major TADs related to gender is sex-determining region Y-related high-mobility group box 9 (SOX9) [17]. It was reported that SOX9 regulated CEACAM1 (carcinoembryonic antigen cell adhesion molecule 1) expression and immune resistance in melanoma [21]. Furthermore, PD-L1 expression appears to be directly or indirectly regulated by several X-linked miRNAs [22]. According to TargetScan 7.1 (http://www.targetscan.org), three miRNAs localized on chromosome X, including miR-106b, miR-20b, and miR-513, are repressors of PD-L1 by direct binding to their 3′UTRs. PD-L1 transcription is induced by hypoxia-inducible factor-1α (HIFα) and signal transducer and activation of transcription-3 (STAT3) factors, directly acting on its promotor [23]. miR-20b can target both HIF-1a and STAT3 transcription factors [24], thus repressing PD-L1 expression. Therefore, there might be a gene-to-gene enhancing coalition effect among sex and PD-L1 expression. Further investigation to understand the mechanisms underlying the gender disparity and aging in the treatment response for GC is still needed.

In the study of Sasao et al. [7], for patients aged 40 or younger, males were more likely to have tumor located in the lower-third of the stomach than females (30% vs. 5%). In the present study, as shown in Table 1, females were more likely to have tumors in the middle-third stomach than males before propensity-score matching. For the subgroup analysis for younger and older patients, because the number of patients aged 40 or younger is too small for analysis, we choose an age of 65 years or older to categorize aged patients according to the World Health Organization. In the present study, for patients aged younger than 65 years, females were more likely to have tumor located in the middle-third of the stomach than males (46.5% vs. 37.6%), while tumors in the lower-third of the stomach were more common in males than females (44.3% vs. 37.6%). For patients aged 65 or older, males were more likely to have tumor located in the upper-third of the stomach (20.6% vs. 10.8%). Based on our results and others, it seems that the tumor location of GC was associated with both sex and age.

Regarding the physiological gastrointestinal motility, for liquid diet, there was no significant difference between sexes; however, for solid diet, the gastric emptying diet is significantly longer in females than males [25]. Pre-menopausal females have significantly longer gastric emptying time than males for solid diet [26]. For post-menopausal females, the gastric emptying time decreases and becomes similar to that in males [27]. The gastric emptying time increased with age in the women, while the gastric emptying time remained almost unchanged with age in the males [28]. In addition to gender, age, and the type of meal, patient statue and the size of stomach and diabetic mellitus may also affect gastric motility. The possible impact on the gastric emptying time should be evaluated in separate male and female subpopulations.

In our hospital, there has been a case manager in charge of contacting gastric cancer patients for more than 30 years. Consequently, most patients receive regular follow-up at our hospital. Our previous study demonstrated that approximately 90% of gastric cancer recurrences occurred within 3 years after surgery [29]. In the present study, only 5.8% of our patients had follow-up time shorter than 3 years, we believe that the small percentage of patients have little impact on the tumor recurrence rate in the present study.

Because this study is a retrospective study with single center data, this study has limitations. Consequently, we performed propensity-score matching to minimize selection bias. GC, a multifactorial disease, is easily associated with dietary culture and economical levels. Diet with high fat/salt/nitrogen, a history of infection with Helicobacter pylori or Epstein-Barr virus, tobacco use, immune system, and genetic mutations caused by chronic inflammation are risk factors for cancer development [30–34]. In the present study, we could not analyze the influence of dietary and economical levels in our GC patients because the data is not available in most patients. Although our results demonstrated higher *PD-L1* expression in males than females, especially in stage III GC, none of our enrolled patients received immunotherapy for tumor recurrence. We could not evaluate the efficacy of immunotherapy in this group of patients. Further randomized controlled trials investigating the use of immunotherapy for adjuvant therapy are required to validate our results and may provide convincing evidence regarding GC treatment in the future.

In conclusion, males were associated with more tumor recurrence and higher *PD-L1* expression than females, especially in stage III GC. Examining *PD-L1* expression is recommended for GC patients with liver metastases, especially aged male patients; immunotherapy may be beneficial for this group of GC patients. Further randomized controlled studies are required to validate our results.

## MATERIALS AND METHODS

### Patients and sample collection

Between January 1992 and December 2013, in total, 2673 GC patients with adenocarcinoma who underwent curative surgery (R0 resection) were enrolled in the present study. Propensity-score matching was performed to minimize selection bias in the comparison of the clinicopathological features between the sexes.

Tumor and normal gastric mucosa tissues were collected and stored in a biobank at our institution. The genetic alterations of 438 GC patients with available tumor and normal gastric mucosa tissues were analyzed. The pathological staging of the GC was performed according to the 8^th^ edition of the American Joint Committee on Cancer (AJCC)/Union for International Cancer Control (UICC) TNM classification system [35].

### Follow-up

Follow-up examinations, including physical examinations, blood tests with measurements of tumor markers, chest radiography, and sonography or computerized tomography scans of the abdomen, were performed every 3 months during the first 3 years after surgery and every 6 months thereafter.

### DNA extraction and analysis of microsatellite instability and genetic mutations

The DNA extraction from the tissue specimens was performed using a QIAamp DNA Tissue Kit and a MinElute Virus Kit (Qiagen, Valencia, CA, USA) according to a previous report [36].

As described in a previous study [12], five reference microsatellite markers, namely D5S345, D2S123, D17S250, BAT25 and BAT26, were used to determine MSI. MSI-high (MSI-H) was defined as ≥ 2 loci of instability with 5 markers, while MSI-low/stable (MSI-L/S) was defined as one unstable locus or no MSI loci.

A MassARRAY system (Agena, San Diego, CA, USA) was used to identify 68 mutation hotspots in 8 GC-related genes (*TP53*, *ARID1A*, *PTEN*, *PIK3CA*, *AKT1*, *AKT2*, *AKT3*, and *BRAF*) [36]. *PI3K/AKT* pathway genetic mutations was defined as mutations identified in *PIK3CA*, *PTEN*, *AKT1*, *AKT2*, or *AKT3*.

### Immunohistochemical (IHC) staining for PD-L1

IHC staining was performed to detect *PD-L1* expression using *PD-L1* IHC 22C3 pharmDx kit on a Dako ASL48 platform [13]. The combined positive score (CPS) was calculated, and a CPS score >1 was defined as positive expression of *PD-L1*.

### Propensity-score matching strategy

As shown in Table 1, to minimize selection bias, propensity-score matching was performed based on logistic regression modeling of seven covariates (age, tumor location, gross appearance, Lauren’s classification, lymphovascular invasion, adjuvant chemotherapy, and pathological TNM stage) to balance the potential confounders between the males and females. A 1:1 ratio matching of males and females was applied. A specific caliper width equal to 0.1 standard deviation was used.

### Statistical analysis

IBM SPSS Statistics 25.0 (IBM Corp., Armonk, NY, USA) was used for the statistical analyses. A χ^2^ test with Yates correction or Fisher’s exact test was used to compare the categorical data. The OS was defined from the date of surgery to the date of death or the last follow-up, while DFS was defined as the length of time after surgery during which the patient was alive without GC recurrence. The Kaplan–Meier method was used for the survival analysis of the OS and DFS. A multivariate analysis with Cox proportional hazards models was performed to analyze the independent prognostic factors of the OS and DFS. A *P* value < 0.05 was defined as statistically significant.
